# Supplementary material for: Association of cesarean section and infectious outcomes among infants at 1 year of age: Logistic regression analysis using data of 104,065 records from the Japan Environment and Children’s Study
Source: PLoS One. 2024 Feb 21;19(2):e0298950. doi: 10.1371/journal.pone.0298950 (PMC10880998; doi:10.1371/journal.pone.0298950)
Supplement: Attachment — Submitted filename: 20231206_Responses to the Reviewers_R1_PLOS ONE_Final.docx [file pone.0298950.s001.docx]

Responses to the Reviewers

Manuscript ID: PONE-D-23-20377

We wish to re-submit the manuscript titled “Association of cesarean section and infectious outcomes among infants at 1 year of age: Logistic regression analysis using data of 104,065 records from the Japan Environment and Children’s Study.”

We thank the reviewers for their insightful review and valuable comments, which have helped us improve our manuscript significantly. We have addressed the major concerns of the reviewers and provided a point-by-point response to the reviewers’ comments below.

Reviewer #1: In this manuscript, the authors investigated the association between caesarean section and infectious diseases in children in Japan using the Japan Environment and Children's Study (JECS). The manuscript is well written and well organized. This study is important and interesting because there is controversy about this association. However, I found some problems as listed below, which, if addressed, will improve the manuscript.

Major
1) Study design, measurement and reporting of association
On page 28, line 253, the authors describe this study as a "prospective and nationwide cohort design". The study design should be clearly stated in the title, abstract and material and methods. If this is a prospective cohort study, why did the authors report the measure of association using odds ratio rather than relative risk?

**Author response:** Thank you for your valuable comment. Prospective cohort studies have the advantage of using a variety of measures. However, relative risk cannot be used in case-control studies. Therefore, the odds ratio is used as an approximation of the relative risk in case-control studies. In this cohort prospective study, we investigated the independent association between cesarean sections and infectious diseases. Therefore, we performed a logistic regression analysis to control for confounding factors and calculated the odds ratios. We have revised the text of the manuscript as follows:

Page 3, lines 30–31

This study used data from the Japan Environment and Children’s Study which is a prospective, nationwide, government-funded birth cohort study.

Page 5, lines 75–76

We used data from the JECS, a prospective nationwide government-funded birth cohort study [20].

2) Outcome description
It was often difficult to understand what population the authors wanted to know about infections. Of course, I could imagine from the title and context that the authors wanted to focus on infectious diseases in children from birth to one year, but this information should be clearly explained. On page 29, line 273-274, the authors concluded that "further studies are needed to evaluate the role of caesarean delivery in the development of neonatal infectious diseases", and this caused me further confusion because this article was not only about neonatal infectious diseases.

**Author response:** We apologize for this confusion. We focused on infectious diseases in infants. We have revised the text of the manuscript as follows:

Page 22, lines 250–252

Further studies are needed to evaluate the role of cesarean delivery in the development of infectious diseases in infants.

3) Validity of key information
Although the authors explain in the limitation about the potential underestimation of infectious disease, but this should be discussed further. From the "Data collection" section, all information after the neonatal period was from self-reported questionnaires. The information on infectious diseases is essential in this study. The authors should explain the details of the validity of the information collected by questionnaires and how the result could be influenced There was 'under-reporting' as described by the authors.
(It may be useful to cite an article explaining the validation of the JECS, if available)

**Author response:** Thank you for your valuable comments.

In the JECS plan, the follow-up rate was to be 80% or higher at the end of the follow-up period. It is highly commendable that the follow-up rate has remained high (93.6% on average nationwide) as of September 2022. Moreover, one of the most important issues in making the results of the JECS survey more reliable is maintaining a high participant questionnaire recovery rate. Eighty percent retention is the target for the JECS. The average recovery rate for 1-year-olds was 91.4%. We have revised the text of the manuscript as follows:

Page 21-22, lines 240–244

Despite these limitations, our study evaluated data from a large, nationwide prospective birth cohort study that has maintained high follow-up and questionnaire response rates [20]; therefore, our study provides strong evidence against an association between cesarean delivery and infectious diseases.

Minor
1) Page 13, line 113-114
The word "morbidity" should be replaced by another word if the authors meant simple incidence of infectious disease rather than death.

**Author response:** Thank you for your valuable comment. We used “morbidity” to mean the incidence of infectious diseases. We would use “mortality” if we wanted to refer to death.

2) Page 17, lines 176-179.
It is appropriate to describe the phi coefficient here in the results section, but its assessment should be discussed in the discussion section.

**Author response:** Thank you for your valuable comment. We have revised the text of the manuscript as follows:

Page 19, lines 189–193

Cesarean delivery was significantly associated with an increased risk of infection. However, the phi coefficient between the two groups was 0.0089, and the difference was interpreted as very small. Such a small difference was considered significant because of the large sample size and detection power of this study.

Reviewer #2: This report uses data from the Japan Environment and Children's Study to show that there is no association between cesarean section and infant infections. This is a valuable report that shows the situation in Japan based on large amounts of data, but some corrections are needed.

Major comments
1. Regarding infections up to 1 year of age, are confounding factors whether the child has an underlying disease or whether the cesarean section was elective or emergency? Other papers report investigations limited to the elective cesarean section. I think the author should discuss the reason for not including it as an adjustment factor or list it in the limitations section.

**Author response:** Thank you for your valuable comment. We have added the following text and reference to the revised manuscript:

Page 20, lines 219–222

These mechanisms may affect elective cesarean deliveries more than emergency cesarean deliveries because emergency cesarean deliveries often occur after the onset of labor, potentially resulting in exposure to vaginal microflora and maternal and fetal stress [23].

Page 21, lines 237–238

Third, we did not distinguish between emergency and elective cesarean sections.

References, Page 26, lines 340-342

23. Rusconi F, Zugna D, Annesi-Maesano I, Baïz N, Barros H, Correia S, et al. Mode of Delivery and Asthma at School Age in 9 European Birth Cohorts. Am J Epidemiol. 2017; 185: 465-473.

2. L206-238 How are these sentences related to the results of the authors’ study? I believe it is simply explaining the efficacy of vaccines and the epidemiology of each infectious disease.

**Author response:** Thank you for your valuable comment. We have removed lines 206–238 and references 23–34 from the text.

Minor comments
1. L79-81 Why do authors also classify by region (e.g., Koshin and South Kyusyu/Okinawa) instead of the prefecture name? Is it differentiated based on the distribution of the number of cases? Please explain.

**Author response:** Thank you for your valuable comment. To ensure generalizability and the ability to extrapolate the JECS results to the Japanese population, 15 regional centers covering a wide geographical area were selected. The urbanization and land development of the study locations are diverse. The urbanization status ranged from urban and suburban to rural, and the land development purposes ranged from agricultural and fishery to commercial and industrial uses. Regional centers were selected through a competitive process in which universities and other research institutions were invited to submit proposals for covered areas and populations, recruitment methods, organizational structures, regional liaison, and resources. Each regional center consists of one or more study areas. The population of the selected study areas ranged from 130,000 to 600,000. Assuming a birth rate of 1% in study areas, each regional center will have 1,300 to 6,000 annual births, or 4,400 annual births on average. The JECS aims to cover half of the births in the area. The selected regional centers are required to recruit 3,000 to 9,000 pregnant women in three years, totaling 100,000 participants from the 15 regional centers. We have added the following text to the revised manuscript:

Page 5, lines 81–83

Each regional center consisted of one or more study areas to ensure generalizability and the ability to extrapolate the results of the JECS to the Japanese population.

2. L109-112 Is there a definition for each infectious disease?
Furthermore, the reviewer could not understand the meaning of the statement “based on the doctor’s self-reported diagnoses and information given participants’ questionnaires.” Does the database also include the data from a questionnaire survey among doctors regarding their diagnosis? Or did the family select each disease on a questionnaire based on the doctor's diagnosis?

**Author response:** Thank you for your valuable comment. The family selected each disease on the questionnaire based on the doctor’s diagnosis. We have revised the text of the manuscript as follows:

Page 7, 110–113

Central nervous system infection (CNSI), OM, upper respiratory tract infection (URTI), LRTI, gastrointestinal infection (GI), and urinary tract infection (UTI) were assessed based on the information given in the participants’ questionnaires when the children were one year old (C1Y data).

3. L117-122 As in the table, please also indicate whether each question is perinatal socioeconomic or postnatal confounding in the Method section.

**Author response:** Thank you for your valuable comment. We have revised the text of the manuscript as follows:

Page 8, lines 117–124

We considered the following factors as possible confounders in the regression analyses: perinatal factors including maternal age at pregnancy, parity, sex, preterm, small for gestational age (SGA), maternal allergy, maternal active or passive smoking during pregnancy, and maternal drinking during pregnancy; socioeconomic factors including maternal education periods, annual family income, and marital status; and postnatal factors including breastfeeding at six months, pet ownership, passive smoking exposure of infants after birth, children’s allergy, sibling, nursery, and vaccination status.

4. L175-176 Why not did the authors mention the results of aOR*1 regarding the relationship between overall infections and cesarean sections, as well as the results of aOR*2? Please let me know if there is a reason.

**Author response:** Thank you for your valuable comment. We have revised the text of the manuscript as follows:

Page 11, lines 177–181

After adjusting for perinatal and socioeconomic confounding factors, cesarean delivery was significantly associated with an increased risk of infection (aOR = 1.04; 95% CI, 1.00–1.09). After adjusting for perinatal, socioeconomic, and postnatal confounding factors, cesarean delivery was significantly associated with an increased risk of infection (aOR = 1.05; 95% CI, 1.00–1.09).

5. L188-200 This section introduces articles from other countries that reported a relationship between cesarean sections and infectious diseases, unlike the authors’ study, and describes "These results are inconsistent with our results." Thus, reference 19 reported that there is no relationship with infectious diseases, similar to the author's research, so I think the sentences (L188-191) are inappropriate for this part of the article.

**Author response:** Thank you for your valuable comment. We have revised the text of the manuscript as follows:

Page 19, line 206

These results are controversial.

6. L268 Based on the results of this research, I think "infant" is correct rather than "neonatal."

**Author response:** Thank you for your valuable comment. We have revised the text of the manuscript as follows:

Page 22, lines 244–246

If cesarean delivery has clinical benefits, it should not be avoided because of the risk of infection in the infant.

Journal Requirements:

When submitting your revision, we need you to address these additional requirements.

1. Please ensure that your manuscript meets PLOS ONE's style requirements, including those for file naming. The PLOS ONE style templates can be found at

<https://journals.plos.org/plosone/s/file?id=wjVg/PLOSOne_formatting_sample_main_body.pdf> and

<https://journals.plos.org/plosone/s/file?id=ba62/PLOSOne_formatting_sample_title_authors_affiliations.pdf>

**Author response:** We have ensured that our manuscript meets PLOS ONE’s style requirements.

2. Note from Emily Chenette, Editor in Chief of PLOS ONE, and Iain Hrynaszkiewicz, Director of Open Research Solutions at PLOS: Did you know that depositing data in a repository is associated with up to a 25% citation advantage (https://doi.org/10.1371/journal.pone.0230416)? If you’ve not already done so, consider depositing your raw data in a repository to ensure your work is read, appreciated and cited by the largest possible audience. You’ll also earn an Accessible Data icon on your published paper if you deposit your data in any participating repository (<https://plos.org/open-science/open-data/#accessible-data>).

**Author response:** The data are unsuitable for public deposition due to ethical restrictions and Japan’s legal framework. The Act on the Protection of Personal Information (Act No. 57 of May 30, 2003; amended on September 9, 2015) prohibits the public deposition of data containing personal information. The Ethical Guidelines for Medical and Health Research Involving Human Subjects enforced by the Japan Ministry of Education, Culture, Sports, Science, and Technology and the Ministry of Health, Labor, and Welfare also restrict the open sharing of epidemiological data. All inquiries regarding data access should be sent to jecs-en@nies.go.jp. Dr. Shoji F. Nakayama of the JECS Program Office, National Institute for Environmental Studies, is responsible for handling the inquiries sent to this e-mail address.

3. We noticed you have some minor occurrence of overlapping text with the following previous publication(s), which needs to be addressed:

- http://dx.doi.org/10.1136/bmjopen-2017-017086

In your revision ensure you cite all your sources (including your own works), and quote or rephrase any duplicated text outside the methods section. Further consideration is dependent on these concerns being addressed.

**Author response:** In this publication, “Associations of caesarean delivery and the occurrence of neurodevelopmental disorders, asthma or obesity in childhood based on Taiwan birth cohort study” the authors investigated the association between cesarean sections and neurodevelopmental disorders, asthma, or obesity in children in Taiwan using the Taiwan Birth Cohort Study (TBCS). We investigated the association between cesarean sections and infectious diseases in children in Japan using the Japan Environment and Children's Study (JECS). The outcomes differed between our study and this previous study. Therefore, we did not cite this publication. The entire text has been carefully read. However, we could not find any text overlapping with this publication.

4. We note that you have indicated that data from this study are available upon request. PLOS only allows data to be available upon request if there are legal or ethical restrictions on sharing data publicly. For more information on unacceptable data access restrictions, please see <http://journals.plos.org/plosone/s/data-availability#loc-unacceptable-data-access-restrictions>.

In your revised cover letter, please address the following prompts:

a) If there are ethical or legal restrictions on sharing a de-identified data set, please explain them in detail (e.g., data contain potentially sensitive information, data are owned by a third-party organization, etc.) and who has imposed them (e.g., an ethics committee). Please also provide contact information for a data access committee, ethics committee, or other institutional body to which data requests may be sent.

b) If there are no restrictions, please upload the minimal anonymized data set necessary to replicate your study findings as either Supporting Information files or to a stable, public repository and provide us with the relevant URLs, DOIs, or accession numbers. For a list of acceptable repositories, please see <http://journals.plos.org/plosone/s/data-availability#loc-recommended-repositories>.

We will update your Data Availability statement on your behalf to reflect the information you provide.

**Author response:** The data are unsuitable for public deposition due to ethical restrictions and Japan’s legal framework. The Act on the Protection of Personal Information (Act No. 57 of May 30, 2003; amended on September 9, 2015) prohibits the public deposition of data containing personal information. The Ethical Guidelines for Medical and Health Research Involving Human Subjects enforced by the Japan Ministry of Education, Culture, Sports, Science, and Technology and the Ministry of Health, Labor, and Welfare also restrict the open sharing of epidemiological data. All inquiries regarding data access should be sent to jecs-en@nies.go.jp. Dr. Shoji F. Nakayama of the JECS Program Office, National Institute for Environmental Studies, is responsible for handling the inquiries sent to this e-mail address.
