# Supplementary material for: Association of cesarean section and infectious outcomes among infants at 1 year of age: Logistic regression analysis using data of 104,065 records from the Japan Environment and Children’s Study
Source: PLoS One. 2024 Feb 21;19(2):e0298950. doi: 10.1371/journal.pone.0298950 (PMC10880998; doi:10.1371/journal.pone.0298950)
Supplement: Attachment — Submitted filename: Responses to the Reviewers_R2_Final.docx [file pone.0298950.s002.docx]

Responses to the Reviewers

Manuscript ID: PONE-D-23-20377

We wish to re-submit the manuscript titled “Association of cesarean section and infectious outcomes among infants at 1 year of age: Logistic regression analysis using data of 104,065 records from the Japan Environment and Children’s Study.”

We thank the reviewers for their insightful review and valuable comments, which have helped us improve our manuscript significantly. We have addressed the major concerns of the reviewers and provided a point-by-point response to the reviewers’ comments below.

Reviewer #1: Although most of the comments were reviewed and corrected appropriately, this article still has ambiguous parts.

Major

1. Study design
For my comment about the study design in the first revision, the authors answered that this study is a "case-control study". However, the authors did not mention this anywhere in the manuscript and this is confusing for readers. From my understanding, this is a nested case-control study using an existing cohort. The section on "Study design" does not explain the study design of this study; this is only an explanation of the JECS. The study design of 'this study' should be clarified. Additionally, please clearly describe the definition of case and control.

**Author response:** We apologize for this confusion. This study is a cross-sectional study because it used data obtained at a single time point, divided participants into two groups based on exposure, and compared the outcomes in both groups. We used the JECS dataset and investigated the association between cesarean delivery and the development of infectious diseases in infants. We performed multiple logistic regression analyses to determine the risk of infectious diseases associated with cesarean delivery by eliminating confounding factors and calculating odds ratios. We have revised the text of the manuscript as follows:

Page 3, lines 30–32

This study is a cross-sectional study. We used data from the Japan Environment and Children’s Study, which is a prospective, nationwide, government-funded birth cohort study.

Page 5, lines 75–78

This study is a cross-sectional study. We used data from the JECS and investigated the association between cesarean delivery and the development of infectious diseases in infants. The detailed design of the JECS, a prospective nationwide government-funded birth cohort study, has been previously described [20].

Page 7, lines 113–117

Information about infectious diseases was obtained from the C1Y response to “Has your child ever been diagnosed by a doctor with the following infections?” The following diseases were targeted: central nervous system infection (CNSI), OM, upper respiratory tract infection (URTI), LRTI, gastrointestinal infection (GI), and urinary tract infection (UTI).

Page 7, lines 120–122

The mode of delivery was divided into cesarean sections and vaginal deliveries based on medical record transcripts in the Dr0m data. Participants were assigned to the cesarean or vaginal delivery group.

2. Sample size calculation
How did the authors estimate the sample size? Did the authors try to do matching cases and controls to increase the power?
In a nested case-control study, controls are selected by incidence density sampling or sampling from baseline subcohort. Either way, random sampling for controls is used.
It seems that the authors used all cases and all controls available in the main analysis, but how will the result be affected if they used adequate sample size calculation?

**Author response:** Thank you for your valuable comments. This study is a cross-sectional study. We apologize for this confusion. The ideal sample size has an event-predictor ratio of 10:1 or greater. For each explanatory variable in a multivariate analysis, there should be at least 10 events. This was not a problem in this study because of its large sample size. We have added the following text to the manuscript:

Page 20, lines 199–201

Moreover, the ideal sample size has an event-predictor ratio of 10:1 or greater. Therefore, this was not a problem in this study because of its large sample size.
